# Vertical stratification of plant–pollinator interactions in a temperate grassland

**DOI:** 10.7717/peerj.4998

**Published:** 2018-06-22

**Authors:** Jan Klecka, Jiří Hadrava, Pavla Koloušková

**Affiliations:** 1 Czech Academy of Sciences, Biology Centre, Institute of Entomology, České Budějovice, Czech Republic; 2 Department of Zoology, Faculty of Science, Charles University, Prague, Czech Republic

**Keywords:** Pollination, Plant–pollinator interactions, Entomology, Foraging biology, Plant reproduction, Bees

## Abstract

Visitation of plants by different pollinators depends on individual plant traits, spatial context, and other factors. A neglected aspect of small-scale variation of plant–pollinator interactions is the role of vertical position of flowers. We conducted a series of experiments to study vertical stratification of plant–pollinator interactions in a dry grassland. We observed flower visitors on cut inflorescences of *Centaurea scabiosa* and *Inula salicina* placed at different heights above ground in two types of surrounding vegetation: short and tall. Even at such a small-scale, we detected significant shift in total visitation rate of inflorescences in response to their vertical position. In short vegetation, inflorescences close to the ground were visited more frequently, while in tall vegetation, inflorescences placed higher received more visits. Moreover, we found major differences in the composition of the pollinator community on flowers at different heights. In a second experiment, we measured flower visitation rate in inflorescences of *Salvia verticillata* of variable height. Total flower visitation rate increased markedly with inflorescence height in this case. Data on seed set of individual plants provide evidence for a corresponding positive pollinator-mediated selection on increased inflorescence height. Overall, our results demonstrate strong vertical stratification of plant–pollinator interactions at the scale of mere decimetres. This may have important ecological as well as evolutionary implications.

## Introduction

Interactions between plants and their pollinators play an important role in the evolution (Grant & Grant, 1965; Bronstein, Alarcón & Geber, 2006; Suchan & Alvarez, 2015) and maintenance of biodiversity (Bascompte et al., 2003; Bascompte, Jordano & Olesen, 2006; Bascompte & Jordano, 2007) in terrestrial ecosystems. However, the presence and frequency of interactions between particular plants and pollinators vary in time (Olesen et al., 2008) and space (Espíndola, Pellissier & Alvarez, 2011; Newman, Manning & Anderson, 2015). Spatial variation in plant–pollinator interactions is observed from continental scales across the entire distributional range of a plant species (Espíndola, Pellissier & Alvarez, 2011), down to small habitat patches and individual plants (Ohashi & Yahara, 1998; Dupont et al., 2014; Akter, Biella & Klecka, 2017). At the smallest scale, the position of an inflorescence in the context of the surrounding vegetation may affect the frequency and identity of flower visitors with consequences for plant reproduction.

Many plants show high levels of phenotypic plasticity. Inflorescence height is thus highly variable at the intraspecific level and may be important in driving visitation of individual plants. However, the importance of inflorescence height is little understood in grasslands, where the vertical distance between different flowers is rarely more than a few decimetres. In communities of multiple plants species, a few observational studies found that different bee species tend to visit flowers at different heights (Gumbert & Kunze, 1999; Hoehn et al., 2008). In addition, in a trait-based analysis of a plant-flower visitor network in a German grassland, Junker et al. (2013) found that inflorescence height was the most important species trait after phenology to explain which plant species were visited by which insects. Studies investigating the effects of inflorescence height at the intraspecific level found that inflorescence height is under significant pollinator-mediated selection (Sletvold, Grindeland & Ågren, 2010; Jiang & Li, 2017; Trunschke, Sletvold & Ågren, 2017), although these studies unfortunately did not include direct observations of flower visitors.

Although our knowledge of vertical stratification of plant-flower visitor interactions in grasslands is limited, even less is known about how the relationship between pollination and inflorescence height is modified by other environmental factors such as the structure of the surrounding vegetation. For example, Sletvold, Grindeland & Ågren (2013) observed significant pollinator-mediated selection for tall *Dactylorhiza lapponica* plants in tall vegetation, while there was no significant selection on plant height in short vegetation. Similarly, in field experiments with *Primula farinosa*, Ehrlén, Käck & Ågren (2002) showed that short plants were more pollen-limited than tall plants and the difference was larger in a habitat with tall vegetation. However, Ågren, Fortunel & Ehrlén (2006) showed that removal of litter and pruning of vegetation around individual *P. farinosa* plants increased their fruit and seed production apparently because of an increase in their nutritional status rather than increased pollination. The role of vegetation height for pollination thus remains unclear. These studies also looked at the topic entirely from the plant’s point of view and did not measure the effects of vegetation height on visitation frequency or pollinator foraging behaviour. Apart from vegetation height, local density of the same or other plant species (Bartkowska & Johnston, 2014), distance from neighbours (Caraballo-Ortiz, Santiago-Valentin & Carlo, 2011), as well as spatial variation in the proportion of different morphs (Toräng, Ehrlén & Ågren, 2006) can also modify the effects of plant height on its reproductive success.

Most previous research focused on the importance of inflorescence height for plant reproduction, while little attention has been devoted to understanding whether and why foraging insects prefer flowers at certain heights. Some insight can be gained from observations of foraging behaviour of individual insects. In honeybees, ‘horizontal movement’ characterized by a tendency of individual bees to fly between plants of a similar height has been reported (Levin & Kerster, 1973; Faulkner, 1976). Preference for flowers at a certain height was demonstrated also in solitary bees (Gumbert & Kunze, 1999; Hoehn et al., 2008) and wasps (Peakall & Handel, 1993). Flying at a constant height may be advantageous from an energetic point of view for optimally foraging flower visitors (Pyke, 1978). Also, flowers close to the ground may be avoided by some insects because their visitation requires the ability to manoeuvre among plant stems, which may be challenging in dense vegetation (Gumbert & Kunze, 1999).

We conducted a set of field experiments in a dry grassland in the Czech Republic to fill in some of these knowledge gaps. Specifically, our aim was to test whether total visitation rate and the composition of flower visitor assemblages depend on inflorescence height and whether the relationship is modified by the height of the surrounding vegetation. Another aim was to test whether inflorescence height is under pollinator-mediated selection in our system. Our field experiments with three species of plants common in dry grasslands in Central Europe showed that visitation rate varied with inflorescence height. Moreover, the relationship differed between different flower visitor taxa and was modified by the height of the surrounding vegetation. We also detected significant increase in seed production with inflorescence height in *Salvia verticillata*.

## Methods

### Field experiments

We conducted two field experiments in a dry grassland near Český Krumlov, in the southern part of the Czech Republic (48°49′28″N 14°18′59″E). The study site is a species rich calcareous grassland on a southwest-facing slope managed by occasional pasture by cows and sheep. The area is state-owned and publicly accessible. No permits were needed for this study.

In the first experiment, we observed visitation of inflorescences of two plant species, *Centaurea scabiosa* and *Inula salicina*, at different heights above ground. To avoid confounding factors, e.g. taller plants having a different size of inflorescences than shorter plants, we used inflorescences cut from plants in the local population. We selected inflorescences of a similar size and general appearance and placed them in 15 ml tubes with water. We attached each tube to a bamboo stick of different length and attached the stick to the ground. This way, we manipulated the height of the inflorescence between 5 and 105 cm above ground. We placed the inflorescences along two short transects, each containing seven inflorescences placed 50 cm apart. One transect was surrounded by short and the other by tall and dense vegetation; the transects were ca. 10 m apart. The area of short vegetation was grazed by cows in the spring, while the area of tall vegetation was not managed. Short vegetation was characterized by most plants <10 cm tall; the average height of inflorescences of all plants growing within 50 cm from the transect in all directions was 7.2 cm (SD = 6.00). Tall vegetation was composed of a dense layer of plants reaching ca. 50 cm; plants growing within 50 cm from the transect had flowers on average 50.1 cm above ground (SD = 14.73). We individually placed seven inflorescences of either *C. scabiosa* or *I. salicina* in each transect at 5, 15, 25, 45, 65, 85, and 105 cm above ground in a randomized order.

We observed visitation of the inflorescences by insects between 10:30 and 16:00 h, for 30 min in each transect, and identified all visitors at the species level or classified them into taxonomical groups with the highest precision we could achieve without capturing the insects. Both transects were observed simultaneously, one person observed each transect. After the 30 min period, we replaced the inflorescences and randomized the order of their vertical position along each transect and took another set of observations. In total, we measured inflorescence visitation in 20 transects in *I. salicina*, 10 in short and 10 in tall vegetation, and 16 transects in *C. scabiosa*, eight in short and eight in tall vegetation. In total, this amounts to 18 h of observations.

In the second experiment, we focused on the effect of inflorescence height for flower visitation rate and its consequences for seed set in *S. verticillata* at the same site. In this case, we did not cut the inflorescences so that we could test whether flower visitation rate varied between inflorescences within the natural limits of their height above ground and to test whether variation in flower visitation rate translated into differences in seed set; i.e. whether female fitness was affected by the vertical position of the inflorescence. We used the following approach to minimize confounding effects, such as taller plants having more resources, different display size, etc. We selected 17 plants of *S. verticillata* with multiple ramets of approximately the same size and with the first several flowers open or with buds ready to start flowering on 18 July 2017. We took advantage of the fact that the inflorescences grow on relatively long and flexible stems. We bent one of them close to the ground, where it was attached to a stick so that the bottom of the inflorescence was positioned just above ground. We made sure that the orientation of the inflorescence remained unchanged. The second stem was attached to another stick so that it reached a maximum height and the third inflorescence was positioned at an intermediate height. This way, the only difference between the inflorescences was their vertical position.

We performed observations of flower visitation in individual *S. verticillata* plants at one of three dates (20 July, 21 July, and 2 August 2017), depending on when they reached the peak of flowering. The three manipulated ramets per plant were observed simultaneously during one 30 min period and filmed using three digital cameras, which gave a total of 25 h of recordings. Afterwards, we measured the height of each inflorescence as a distance of the highest open flower from the ground and counted the number of open flowers. We then watched the recordings and counted and identified all flower visitors. For each visitor, we also counted the number of flowers visited during each inflorescence visit.

We waited for the seeds of *S. verticillata* to ripen and then harvested them on 14 or 24 August 2017 depending on seed development in individual plants. We counted the number of developed seeds and the maximum potential seed set by multiplying the number of flowers by four, which is the number of seeds the plant can produce per flower. We counted the flowers and seeds in individual whorls within each inflorescence separately to gain data on potential differences in percentage seed set along the inflorescence from the lowest to the highest whorl.

As already mentioned, we identified flower-visiting insects without capturing them. Naturally, we could not identify all individuals to the species level, so we classified some of them into higher taxa or categories, such as ‘small solitary bees.’ The most abundant flower visitors were bumblebees, *Bombus* spp., some of which are difficult to identify alive. Fortunately, we have extensive collections from the study site, so we know that there are three species, which we could not distinguish from *Bombus terrestris*, specifically *B. lucorum*, *B. cryptarum*, and *B. magnus*. However, over 85% of individuals of this species group in our collections from this site belong to *B. terrestris*. Similarly, *B. lapidarius* could be confused with *B. confusus* and *B. ruderarius*, but they have rarely been found on the site. A similar level of uncertainty exists in our identification of *B. sylvarum*. The number of potential bumblebee misidentifications during the field observations was thus low and unlikely to confound our results.

### Data analysis

We tested how the total number of visits and the number and proportion of visits by individual flower visitor taxa depended on inflorescence height using generalized additive models (GAM) to account for the non-linear nature of these relationships. The identity of individual plants, each having three ramets manipulated and observed, was included as a random factor in analyses of data from the experiment with *S. verticillata*; i.e. generalized additive mixed models (GAMM) were used in this case. Poisson distribution with overdispersion (quasi poisson distribution) was used for the number of visits, while overdispersed binomial (quasibinomial) distribution was used for data on proportions. These analyses were performed using mgcv 1.8-17 package (Wood, 2006) in R 3.2.3 (R Core Team, 2015).

To gain insights into the effects of inflorescence height on plant fitness, we tested how seed set of individual ramets depended on the number of flowers and the inflorescence height using a generalized linear model. Similarly as in analyses of selection gradients (Lande & Arnold, 1983), we standardized both predictors to have zero mean and unit variance. Partial regression coefficients then allowed us to compare whether reproductive performance (seed set) depended more strongly on the number of flowers or inflorescence height.

## Results

**Figure 1:**
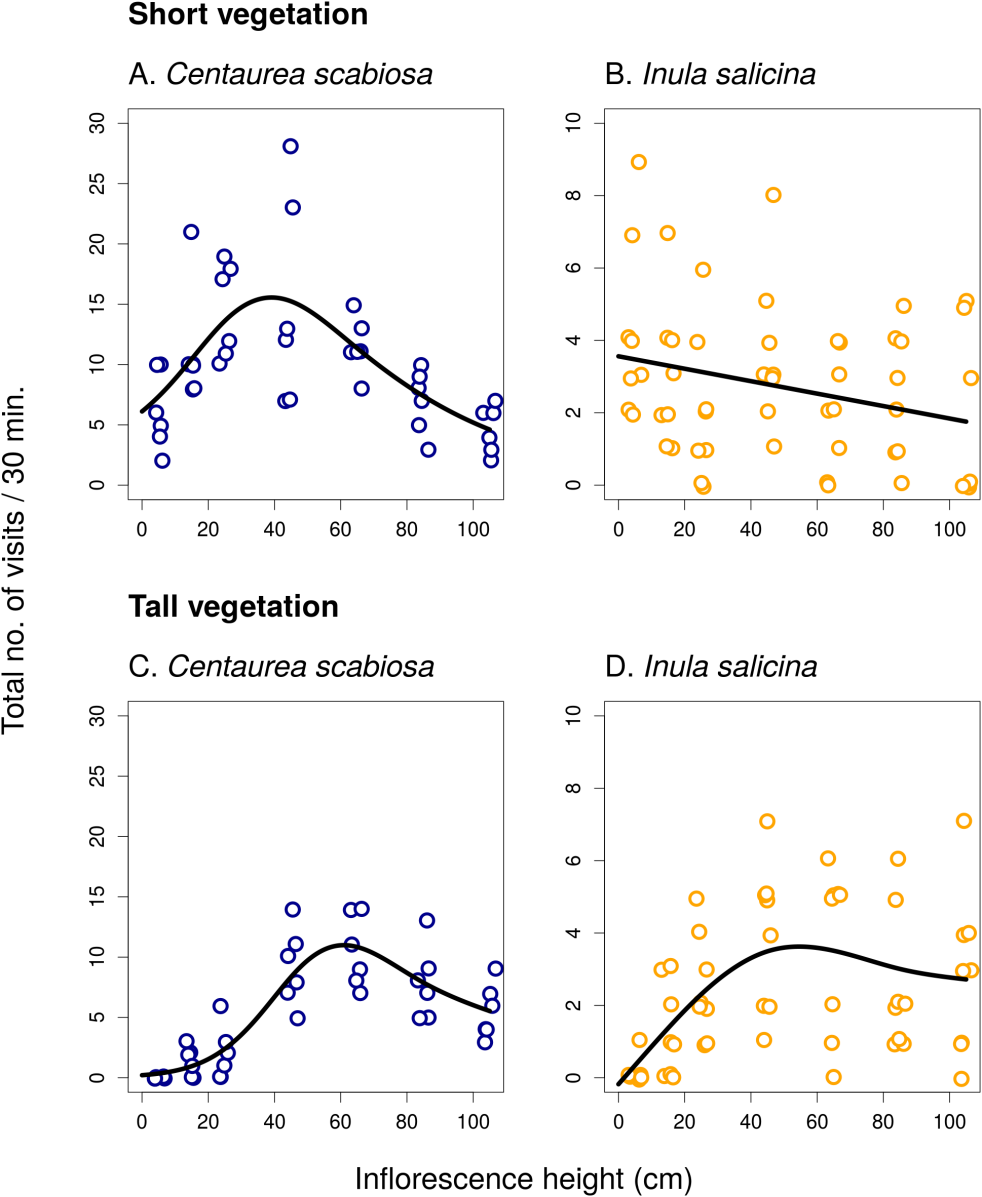
Inflorescence visitation in *Centaurea scabiosa* and *Inula salicina*. Visitation of inflorescences of *Centaurea scabiosa* and *Inula salicina* significantly depended on the vertical position of inflorescences above ground. This relationship was modified by the height of the surrounding vegetation as apparent from the comparison of data from transects surrounded by short (A and B) and tall (C and D) vegetation. A small amount of noise was added to the data in both *x* and *y* direction to make overlapping points visible. Summary of the statistical tests is shown in Table 1.

**Figure 2:**
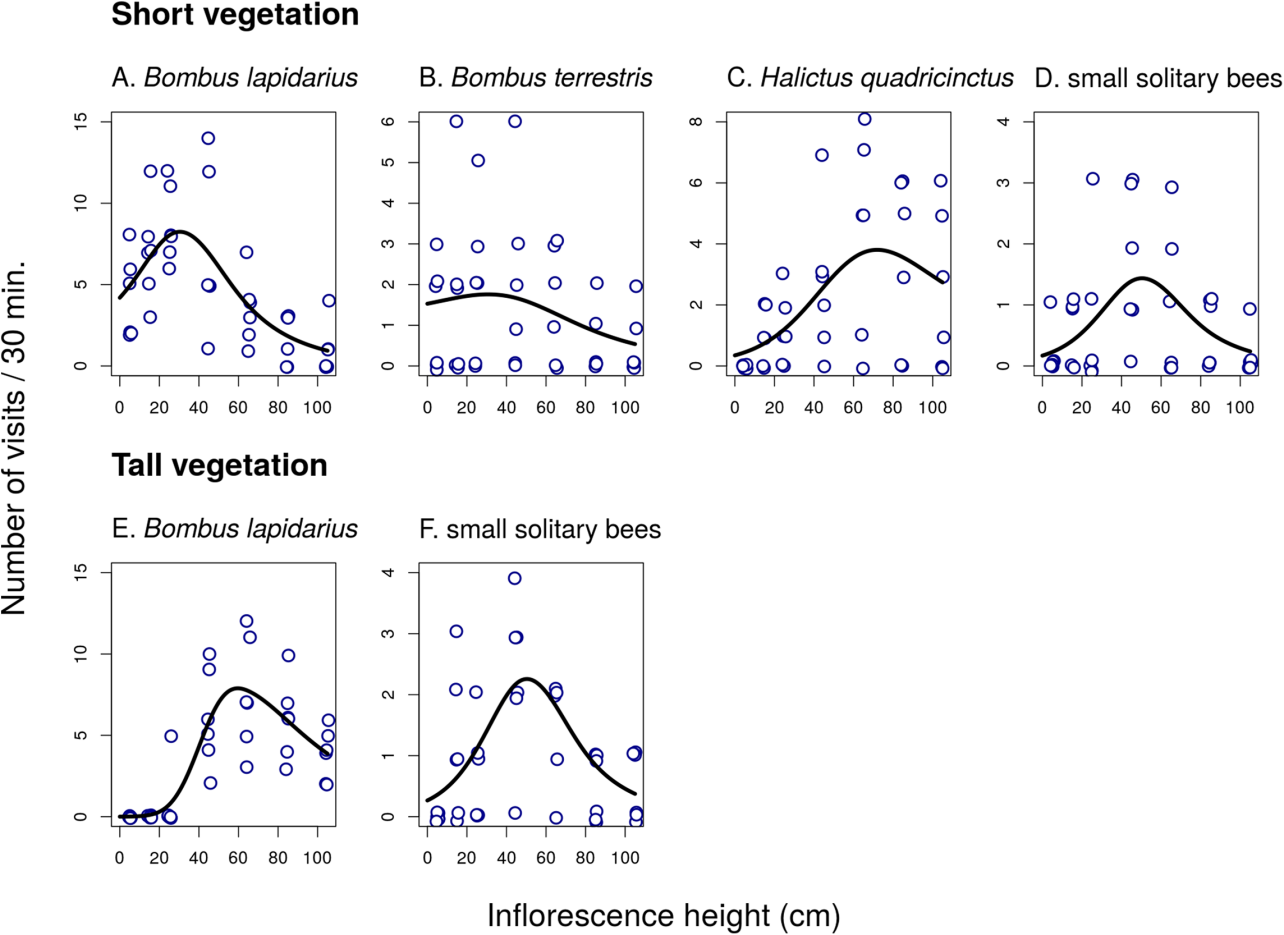
Inflorescence visitation of *Centaurea scabiosa* by the most frequent visitor taxa. The number of visits per 30 min in short (A–D) and tall (E and F) vegetation is plotted. A small amount of noise was added to the data in both *x* and *y* direction to make overlapping points visible. The relationship in *Bombus terrestris* (B) is not statistically significant. Summary of the statistical tests is shown in Table 1.

**Figure 3:**
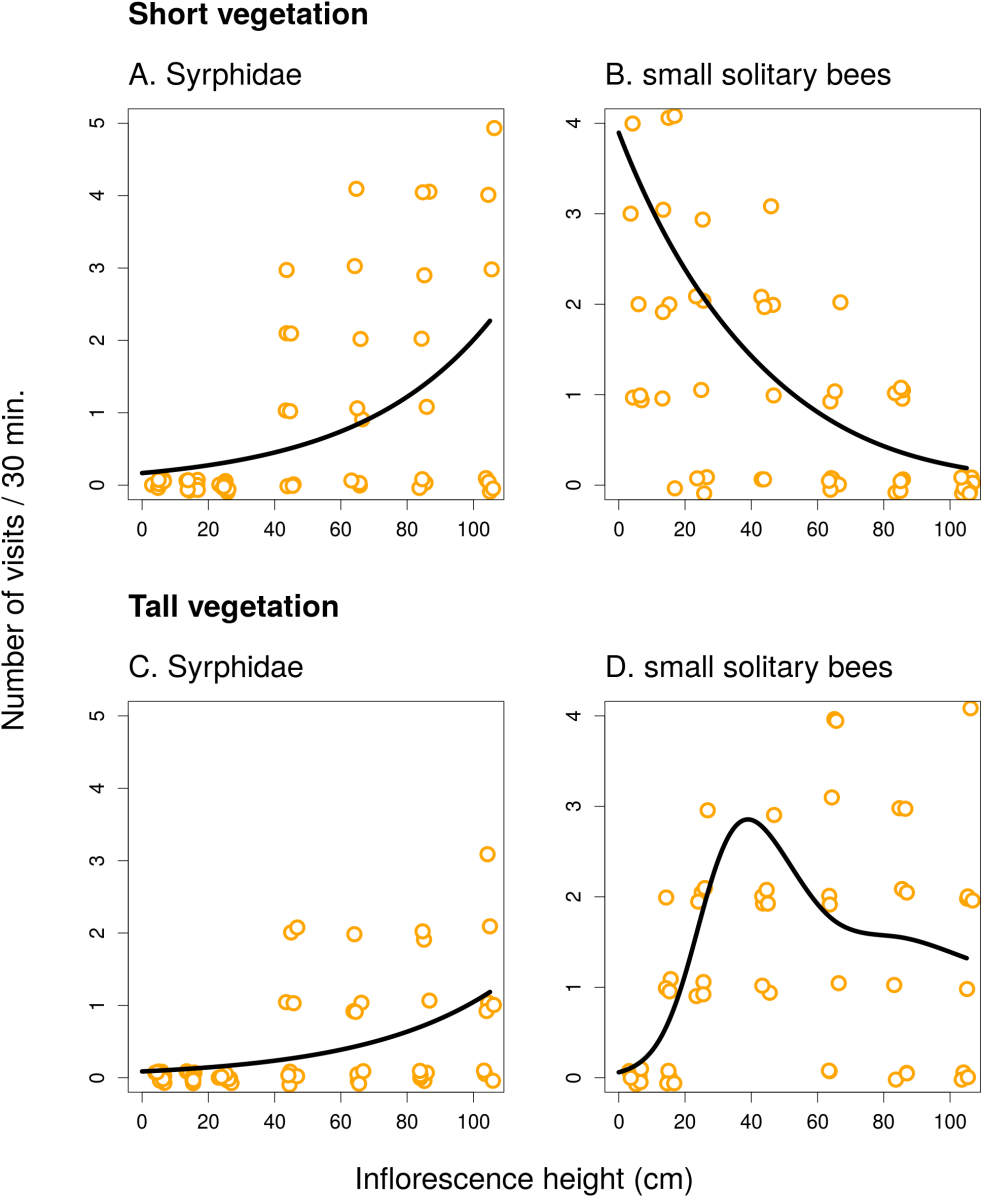
Inflorescence visitation of *Inula salicina* by the most frequent visitor taxa. The number of visits per 30 min in short (A and B) and tall (C and D) vegetation is plotted. A small amount of noise was added to the data in both *x* and *y* direction to make overlapping points visible. Summary of the statistical tests is shown in Table 1.

In the first experiment, we observed a strong, mostly non-linear, dependence of the total visitation rate on inflorescence height in both *C. scabiosa* and *I. salicina* (Fig. 1; raw data: Table S1). Analysis using GAM (Table 1) showed that the relationship was significant in *C. scabiosa* as well as in *I. salicina* in both short and tall vegetation (Table 1). Also, there was a significant difference in the shape of the relationship between total visitation and inflorescence height in short vs. tall surrounding vegetation in both *C. scabiosa* (*F* = 19.27, *P* < 10^−6^) and *I. salicina* (*F* = 12.46, *P* = 3.10 × 10^−5^). Comparison of the results presented in Fig. 1 shows that the difference between short and tall vegetation is mostly that visitation rate of inflorescences of both plant species positioned <50 cm above ground dropped in tall compared to short surrounding vegetation. Moreover, analysis of visitation rate of the most abundant flower visitors showed that different insect species had contrasting height preferences modified by the height of the surrounding vegetation (Figs. 2 and 3; Table 1). Overall, we observed 638 visits (16 taxa) on *C. scabiosa* and 286 visits (13 taxa) on *I. salicina*.

**Table 1:** The effects of inflorescence height and surrounding vegetation height on the number of visits by different insects.

Response	Short vegetation			Tall vegetation			Short vs. tall vegetation	
	edf	*F*	*P*	edf	*F*	*P*	*F*	*P*
**Visits of *Centaurea scabiosa***								
Total visitors	3.00	10.01	3.77 × 10^−6^	3.55	15.21	<1 × 10^−6^	19.27	<1 × 10^−6^
*Bombus lapidarius*	2.80	8.38	4.17 × 10^−5^	3.62	6.15	0.0005	21.32	<1 × 10^−6^
*Bombus terrestris*	1.69	1.92	0.1640	NA	NA	NA	NA	NA
*Halictus quadricinctus*	2.16	6.00	0.0028	NA	NA	NA	NA	NA
Small solitary bees	2.62	8.16	6.20 × 10^−5^	2.62	8.16	6.20 × 10^−5^	0	1
**Visits of *Inula salicina***								
Total visitors	1	5.38	0.0223	2.58	6.27	0.0005	12.46	3.10 × 10^−5^
Small solitary bees	1.32	17.12	4.90 × 10^−6^	3.65	3.75	0.0072	15.39	<1 × 10^−6^
Syrphidae	1.94	9.54	0.0001	1.94	9.54	0.0001	0.04	0.9543

**Notes:**

Summary of results of generalized additive models testing the dependence of visitation of *Centaurea scabiosa* and *Inula salicia* on inflorescence height. Groups of flower visitors which had an insufficient number of observations for analysis were not analysed separately, but were included in the total visitation. edf = estimated degrees of freedom, which gives a measure of the complexity of the shape of the relationship (edf = 1 is a linear relationship). NA = cases when the number of observations was insufficient for analysis. The results are presented graphically in Figs. 1–3.

In *C. scabiosa*, we observed a significant effect of inflorescence height on the number of inflorescence visits by *B. lapidarius*, which preferred mostly inflorescences close to the ground (Fig. 2A; Table 1), *Halictus quadricinctus*, which preferred inflorescence high above ground (Fig. 2C), and small solitary bees, which visited mostly inflorescences at an intermediate height (Fig. 2D). The relationship was not significant in *B. terrestris* (Table 1). A total of two species, *B. terrestris* and *Haliplus quadricinctus*, avoided the area of tall vegetation despite being frequently observed in transects surrounded by short vegetation. On the other hand, *B. lapidarius* was common in both habitats and showed a shift towards inflorescences higher above ground in the transects surrounded by tall vegetation (Figs. 2A and 2E); the relationship between visitation and inflorescence height was significantly different in short and tall vegetation (*F* = 21.31, *P* < 10^−6^). On the contrary, small solitary bees did not shift their visitation (Figs. 2D and 2F).

In *I. salicina*, only two groups of flower visitors were abundant enough for detailed analysis. Hoverflies (Diptera: Syrphidae) visited mostly inflorescences >40 cm above ground and the height of the surrounding vegetation had no effect on the relationship between the number of visits and inflorescence height (Figs. 3A and 3C; *F* = 0.04, *P* = 0.95). On the other hand, small solitary bees favoured inflorescences close to the ground in short vegetation and shifted higher above ground in tall vegetation (Figs. 3B and 3D); the relationship of visitation with inflorescence height was significantly different in short and tall vegetation (*F* = 15.39, *P* < 10^−6^; Table 1).

**Figure 4:**
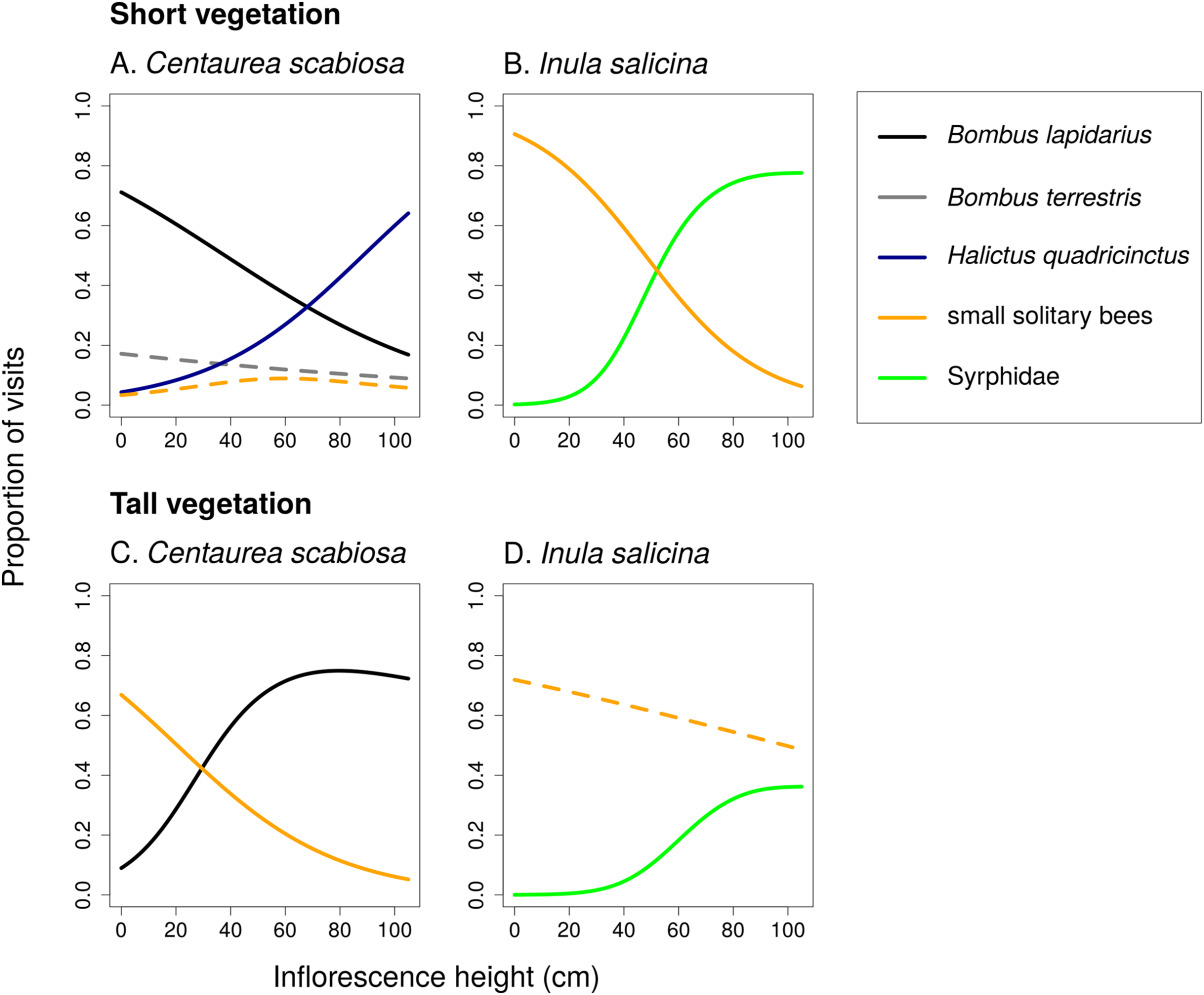
Changes in relative visitation by different insects depending on inflorescence height and vegetation height. Proportions of flower visits attributed to main groups of flower visitors of *Centaurea scabiosa* and *Inula salicina* in transects surrounded by short (A and B) and tall (C and D) vegetation. The relationships were estimated using generalized additive models. A small fraction of visitors belonged to other groups omitted from the analysis because they had very low abundance. Relationships which were not statistically significant are shown in dashed lines. Summary of the statistical tests is shown in Table 2.

Different flower visitors responded to inflorescence height and the height of the surrounding vegetation in a species-specific way (Fig. 4; Table 2). For example, *B. lapidarius* visited mostly inflorescences of *C. scabiosa* positioned close to the ground when the surrounding vegetation was short, but shifted to inflorescences higher above ground when the surrounding vegetation was tall. Visitation of flowers close to the ground surrounded by tall vegetation was then dominated by small solitary bees (Figs. 4A and 4C). The composition of the flower visitor assemblage at a particular height thus differed according to the height of the surrounding vegetation.

**Table 2:** The effects of inflorescence height and surrounding vegetation height on relative visitation by different insects.

Response	Short vegetation			Tall vegetation			Short vs. tall vegetation	
	edf	*F*	*P*	edf	*F*	*P*	*F*	*P*
**Visits of *Centaurea scabiosa***								
*Bombus lapidarius*	1	30.60	<1 × 10^−6^	2.16	4.79	0.0073	14.22	<1 × 10^−6^
*Bombus terrestris*	1	1.28	0.2640	NA	NA	NA	NA	NA
*Halictus quadricinctus*	1	21.21	3.62 × 10^−5^	NA	NA	NA	NA	NA
Small solitary bees	1.72	1.18	0.3045	1	16.02	0.0002	11.871	0.0005
**Visits of *Inula salicina***								
Small solitary bees	1	35.25	<1 × 10^−6^	1	2.16	0.1450	14.78	0.0002
Syrphidae	1.93	15.14	1.46 × 10^−6^	1.93	15.14	1.46 × 10^−6^	1.48	0.2333

**Notes:**

Summary of results of generalized additive models testing the dependence of the proportion of visits attributed to most abundant visitor taxa on inflorescence height in *Centaurea scabiosa* and *Inula salicina*. edf = estimated degrees of freedom, which gives a measure of the complexity of the shape of the relationship (edf = 1 is a linear relationship). NA = cases when the number of observations was insufficient for the analysis. The results are presented graphically in Fig. 4.

**Figure 5:**
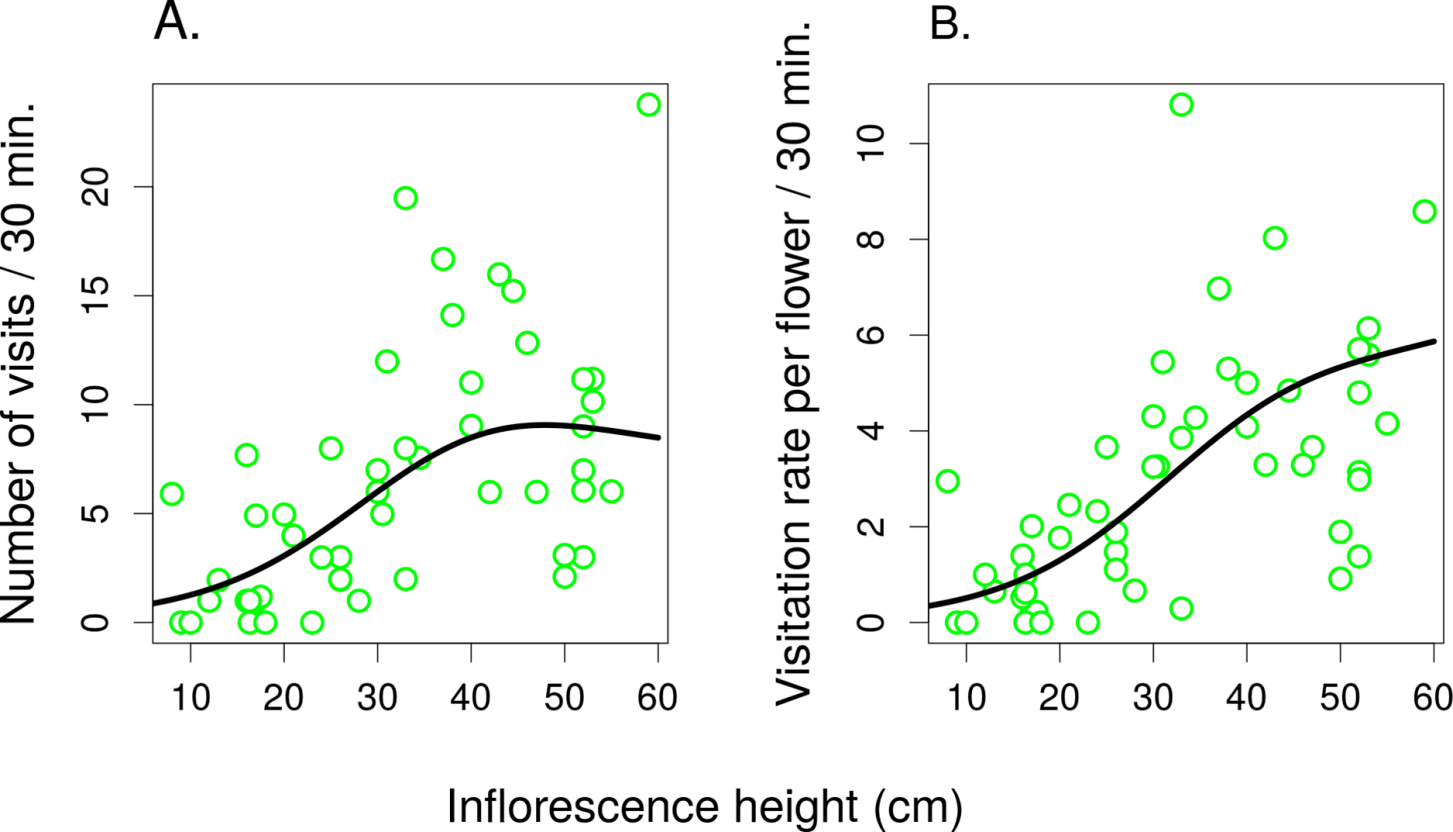
The effect of inflorescence height on visitation of *Salvia verticillata*. The number of visitors per inflorescence (A) and the per-flower visitation rate (B) in inflorescences of *Salvia verticillata* at different heights within the range of heights found naturally at the study site. The inflorescence height refers to the top flower in each inflorescence.

In the second experiment, the total number of flower visitors (Fig. 5A) and the per-flower visitation rate (Fig. 5B) in *S. verticillata* significantly increased with inflorescence height (GAMM; edf = 1.91, *F* = 21.04, *P* = <1 × 10^−6^ and edf = 1.836, *F* = 21.62, *P* = <1 × 10^−6^, respectively) (raw data: Tables S2 and S3). Overall, we observed 300 visits by seven taxa.

**Figure 6:**
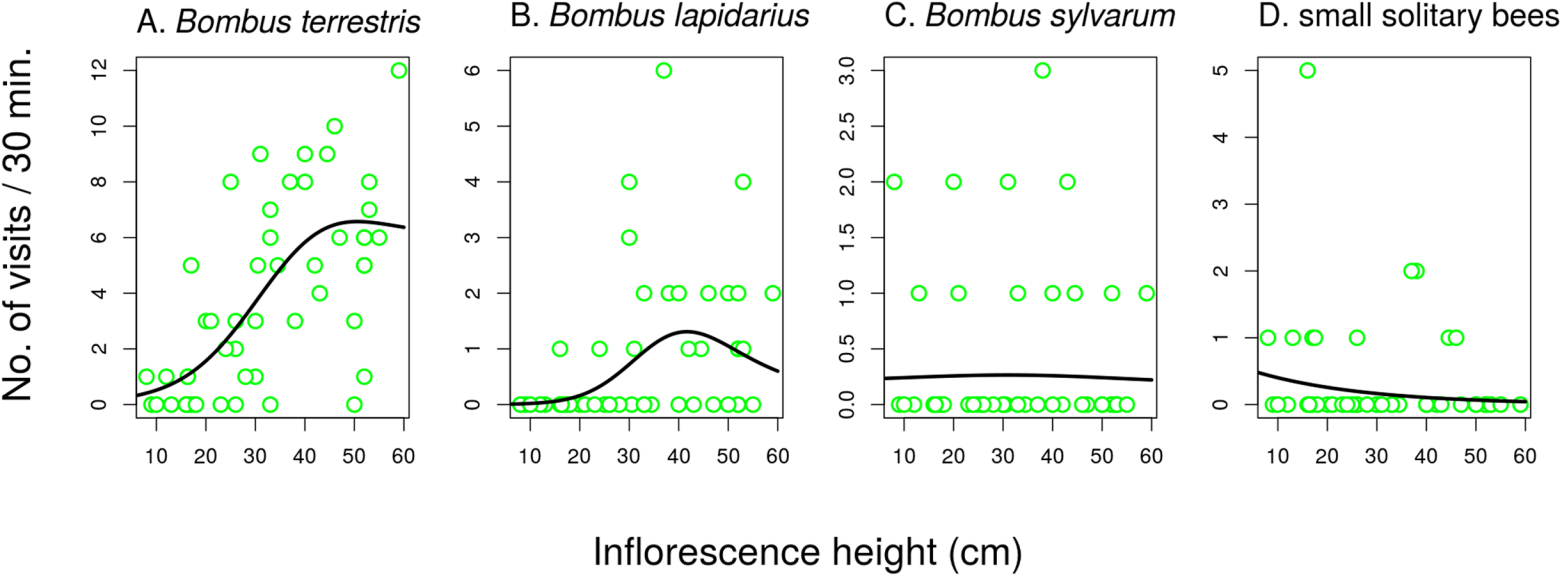
The effect of inflorescence height on visitation of *Salvia verticillata* by different insects. Visitation by the three species of the genus *Bombus* (A–C) and solitary bees (D) was affected by inflorescence height differently. The inflorescence height refers to the top flower in each inflorescence. The relationships are statistically significant except in *Bombus sylvarum* (C). Summary of the statistical tests is provided in Table 3.

**Figure 7:**
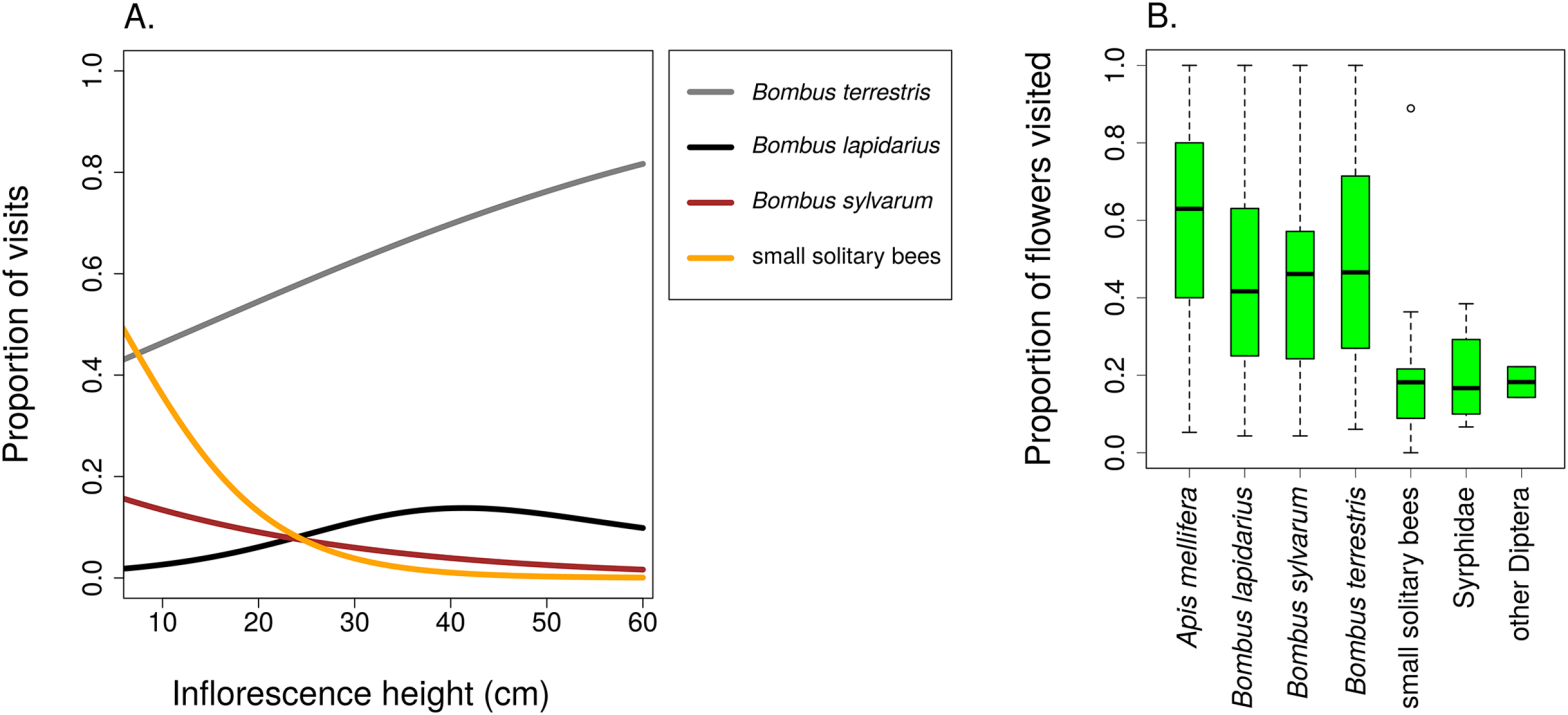
Different insects varied in their contribution to inflorescence visitation and visited different proportions of flowers per inflorescence. (A) Proportions of inflorescence visits attributed to main groups of visitors of *Salvia verticillata* changed significantly in relation to inflorescence height. The relationships are statistically significant except in *Bombus lapidarius*. Summary of the statistical tests is provided in Table 3. (B) Different groups of insects differed in the proportion of flowers visited during a visit to an inflorescence of *Salvia verticillata*. The box and whiskers plot shows the median (horizontal line), interquartile range (box), and 1.5 × SD (whiskers) for each visitor group.

Different groups of flower visitors also showed distinct patterns in their preference for inflorescences of different heights. *B. terrestris* visited mostly the highest inflorescences, followed by *B. lapidarius*, while *B. sylvarum* showed no significant dependence of visitation on inflorescence height and small solitary bees visited mostly inflorescences close to the ground (Fig. 6; Table 3). When expressed as the proportion of visits attributed to individual pollinator groups, our results show that plants with inflorescences closest to the ground were visited equally by *B. terrestris* and small solitary bees (ca. 40% each), followed by *B. sylvarum* (almost 20%) (Fig. 7A; Table 3). On the other hand, visits to inflorescences high above ground were dominated solely by *B. terrestris* (Fig. 7A; Table 3). Different visitors also significantly differed in one aspect of foraging behaviour, namely in the proportion of flowers in an inflorescence probed during a visit (Table 3; GLM, *F* = 5.24, *P* = 3.82 × 10^−5^). *Apis mellifera*, which was excluded from the previous analyses because it was too rare, visited on average over 60% of flowers during one inflorescence visit and the three bumblebee species over 40%. On the contrary, other visitors, which we classified as small solitary bees, Syrphidae, and other Diptera, visited less than 20% of the flowers per inflorescence visit (Fig. 7B).

**Table 3:** The effect of inflorescence height in *Salvia verticillata* on inflorescence visitation.

Response	No. of visits/30 min			Proportion of visits		
	edf	*F*	*P*	edf	*F*	*P*
*Bombus lapidarius*	1.91	8.56	0.0009	1.68	0.89	0.2640
*Bombus terrestris*	1.88	19.28	1.66 × 10^−6^	1	6.90	0.0118
*Bombus sylvarum*	1.21	0.03	0.8700	1	9.93	0.0029
Small solitary bees	1	10.08	0.0026	1	38.48	<1 × 10^−6^

**Notes:**

Summary of results of generalized additive mixed models testing the dependence of the number of visits and the proportion of visits by most frequent visitor taxa on inflorescence height in *Salvia verticillata*. edf = estimated degrees of freedom, which gives a measure of the complexity of the shape of the relationship (edf = 1 is a linear relationship). The results are plotted in Figs. 6 and 7.

**Figure 8:**
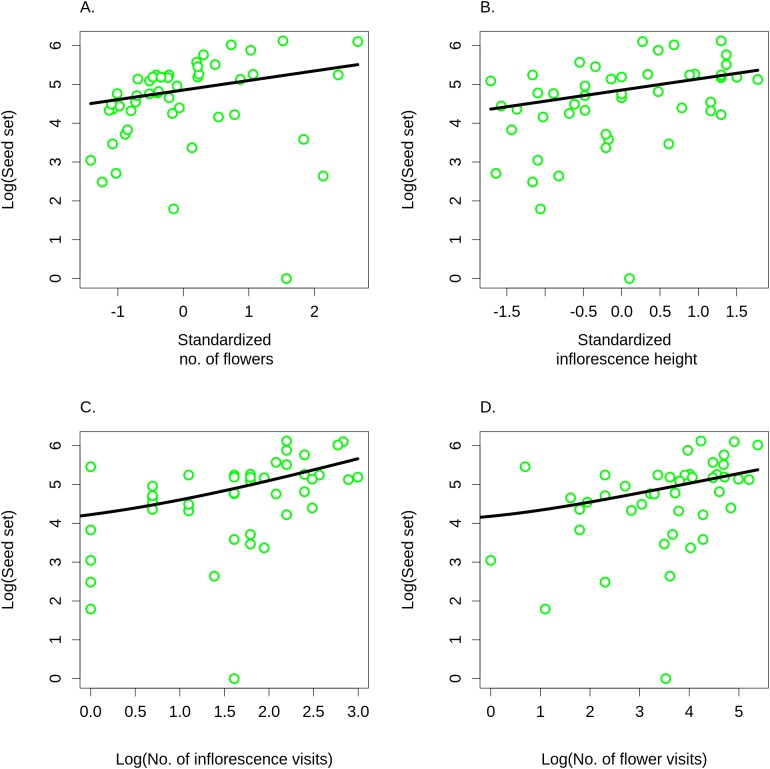
Seed set of *Salvia verticillata*. Seed set of individual ramets of *Salvia verticillata* increased with the number of flowers in the inflorescence (A) and with the inflorescence height (B). Dependence of seed set on inflorescence height could be explained by differences in visitation by pollinators in relation to inflorescence height (see Fig. 5). Seed set increased with visitation measured either as the number of inflorescence visits (C) or as the number of flower visits (D). Including either of these two measures of visitation rate rendered the direct effect of inflorescence height on seed set statistically non-significant.

**Figure 9:**
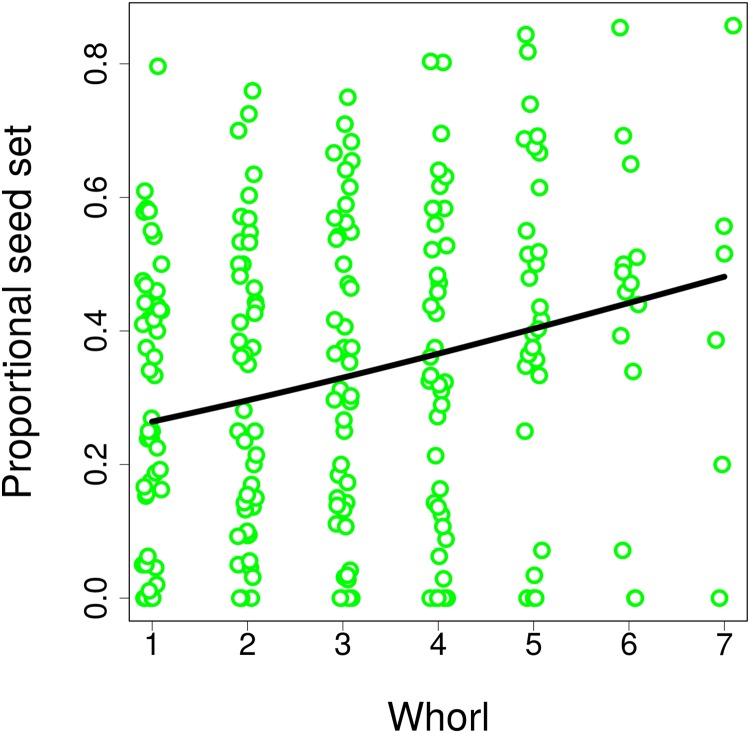
Seed set of individual whorls within inflorescences of *Salvia verticillata*. The proportion of seeds produced relative to the maximum potential seeds set in individual whorls within inflorescences of *Salvia verticillata* increased with the vertical position of individual whorls. Within each inflorescence, the whorl closest to the ground was numbered as 1 and increasing numbers refer to successive whorls higher above ground.

Differences in visitation translated into differences in seed set, which significantly increased with both the number of flowers in an inflorescence (GLM, *F* = 6.21, *P* = 0.0165; Fig. 8A) and with inflorescence height (GLM, *F* = 6.09, *P* = 0.0175; Fig. 8B) (raw data: Table S4). Based on a comparison of partial regression coefficients, seed set depended more strongly on inflorescence height (β = 0.29, SE = 0.101) than on the number of flowers (β = 0.25, SE = 0.089) (both variables were standardized to allow meaningful comparison of regression coefficients). When we included the number of inflorescence visits in the model, there was no longer any significant effect of inflorescence height (*F* = 1.55, *P* = 0.2201), while seed set significantly increased with the number of visits (*F* = 21.46, *P* = 2.98 × 10^−5^; Fig. 8C). We obtained the same results when using the number of flower visits as a predictor (*F* = 9.87, *P* = 0.0030 for the number of flower visits and *F* = 1.76, *P* = 0.1911 for inflorescence height; Fig. 8C), Hence, our results show that increased seed set of taller ramets was driven primarily by increased visitation. The importance of the vertical position of flowers is underscored by the fact that we found a significant increase in the proportional seed set of individual whorls within individual inflorescences when moving from the lowest to the highest whorl (GLM, *F* = 12.80, *P* = 0.0004; Fig. 9).

## Discussion

### The effects of inflorescence and vegetation height on visitation by potential pollinators

In the first experiment, using *C. scabiosa* and *I. salicina* as focal species, total inflorescence visitation peaked approximately at or slightly above the level of the surrounding vegetation in transects surrounded by both short and tall vegetation. These results are consistent with observations that flower visiting bees tend to fly at a specific height and when they leave one inflorescence, they are more likely to fly to another one at a similar height compared to inflorescences lower or higher above ground (Levin & Kerster, 1973; Gumbert & Kunze, 1999). Flowers positioned above the level of a dense layer of vegetation are probably easier to detect and thus attract more visitors (Gumbert & Kunze, 1999). Height preferences of flower visitors may lead to vertical stratification of the plant–pollinator network if different species vary in their behaviour (Roubik, 1993; Gumbert & Kunze, 1999; Ramalho, 2004).

Different responses of individual species or taxonomic groups of flower visitors to inflorescence height, which we observed, mean that inflorescences at different heights varied in the composition of their flower visitor assemblages (Figs. 4 and 7). Plant–pollinator interactions thus appear stratified along the vertical dimension despite the fact that height differences between inflorescences were in the order of mere decimetres. Previous studies on vertical stratification of pollinator communities were conducted mostly in forests where the importance of the vertical dimension is more obvious. It seems there is generally a major difference in the composition of flower visitor communities between the canopy and the understory in tropical (Roubik, 1993; Nagamitsu et al., 1999; Ramalho, 2004) as well as temperate forests (Ulyshen, Soon & Hanula, 2010), probably related to vertical distribution of flowers preferred by different species (Ramalho, 2004). However, vertical stratification of plant–pollinator interactions has been less studied in grasslands where the vertical distances are limited usually to several decimetres. In one of the few available studies (Gumbert & Kunze, 1999), differentiation between visitor communities on flowers below and above the dominant grass layer was observed in a tropical wetland. Similarly, several species of bees visiting pumpkins in an agricultural landscape preferred flowers at different heights (Hoehn et al., 2008). Our results show that vertical stratification of plant-flower visitor interactions may be important also in common temperate grasslands.

An interesting observation is that small solitary bees were the dominant flower visitors close to the ground in all three plant species. We noticed that unlike other flower visitors, they were frequently flying among plant stems close to the ground even in dense vegetation and visiting flowers hidden there, such as flowers of *Rubus* sp., which was growing in parts of the study site. The same pattern was observed by Gumbert & Kunze (1999), who suggested that small bees, which are able to manoeuvre in dense vegetation, may benefit from decreased competition for floral resources because most other flower visitors avoid this microhabitat. This seems to be a likely explanation for our results as well. Behaviour of small solitary bees contrasted with the behaviour of larger species, such as bumblebees, which were flying above the layer of dense vegetation. For example, almost all observations of *B. lapidarius* in tall vegetation were at the height of >40 cm (Fig. 2E).

The effect of flower visitation was thus modified by the height of the surrounding vegetation because most flower visitors avoided flowers within the layer of dense vegetation close to the ground. One bumblebee species, *B. terrestris*, also visited mostly inflorescences close to the ground in short vegetation (Fig. 2B), but almost completely avoided the area with tall vegetation, similarly to *Halictus quadricintus*, a solitary bee, which favoured inflorescences >60 cm above ground (Fig. 2C). Previous studies on the effects of the structure of the surrounding vegetation for flower visitation and plant reproductive success are rare and did not provide clear conclusions. For example, Ågren, Fortunel & Ehrlén (2006) manipulated vegetation height and litter presence around individual plants of *P. farinosa*, and found that litter removal and vegetation pruning increased seed set, especially in short plants. They did not report any data on visitation frequency of flowers in relation to vegetation structure. However, if we assume that higher visitation leads to higher fruit or seed production, our observations of higher visitation of inflorescencences close to the ground in short vegetation exactly mirror these results. In tall vegetation, visitation of both *C. scabiosa* and *I. salicina* peaked higher above ground, which fits the results of Sletvold, Grindeland & Ågren (2013), who observed pollinator-mediated selection for taller inflorescences in tall vegetation but not in short vegetation in a deceptive orchid, *Dactylorhiza lapponica*.

We did not measure the efficiency of different pollinators in the present study, so we cannot infer consequences of the variation of visitation at different heights for the reproductive success of *C. scabiosa* and *I. salicina*. However, data from detailed single visit experiments by other authors demonstrated that different flower visitor species vary in pollen deposition by several orders of magnitude (King, Ballantyne & Willmer, 2013). Hence, it is likely that variation in total visitation rate together with the variation in visitor identity with inflorescence height affects reproductive success of plants in our system. We addressed this question in the second experiment with *S. verticillata*.

### Consequences of inflorescence height for seed set and plant fitness

Observations on *S. verticillata* were constrained by the natural range of inflorescence heights. Unlike previous studies, we collected data both on flower visitation rates by pollinators and on seed set of individual ramets in relation to inflorescence height. Our results are consistent with the hypothesis of positive selection for inflorescence height, which other authors demonstrated in several other plant species (Sletvold, Grindeland & Ågren, 2010, 2013; Jiang & Li, 2017; Trunschke, Sletvold & Ågren, 2017). However, we could also demonstrate that inflorescences positioned higher above ground had higher total flower visitation rates.

Our experimental design allowed us to measure the dependence of seed set on inflorescence height, because we compared similarly looking inflorescences in three ramets per plant (genet), whose height was experimentally adjusted. Vertical position was thus the only apparent difference between the inflorescences. This is important because taller plants usually have higher percentage seed set even when they are hand-pollinated because they have more resources than shorter plants (Andersson, 1996; Červenková & Münzbergová, 2014). Testing for pollinator-mediated selection on inflorescence height thus requires specific experimental designs and is not possible by simply comparing plants of different height. The most frequent approach is to compare selection coefficients for inflorescence height between open-pollinated and hand-pollinated plants (Sletvold, Grindeland & Ågren, 2010; Červenková & Münzbergová, 2014; Jiang & Li, 2017; Trunschke, Sletvold & Ågren, 2017). We used an alternative approach, which allowed us to skip the hand-pollination treatment. We took advantage of the morphology of *S. verticillata*, which creates multiple closely packed, relatively long, and flexible ramets, which can be easily pinned closer to the ground or straightened up without causing damage. For our observations, we selected three ramets with inflorescences of a similar length and general appearance per plant and randomly adjusted their vertical position, so there was no known confounding factor. So, the observed positive correlation between inflorescence height, visitation rate, and seed set can be interpreted as evidence for pollinator-mediated selection on inflorescence height. Nevertheless, some caution is needed, because vertical position of the inflorescence could also affect water transport, which is more difficult to inflorescences higher above ground. Inflorescences at different heights also presumably experienced different levels of shading. In additional, results of our first experiment, where we observed visitation rate on flowers of *C. scabiosa* and *I. salicina* along a wider range of heights, suggest that there may be an optimal height maximising insect pollination depending on the context of the surrounding vegetation.

Flower visitation by different insect species can have different effects on plant fitness not only because different species differ in the number of pollen grains deposited per visit (King, Ballantyne & Willmer, 2013), but also because they differ in the relative frequency of movements between flowers on the same plant and between different plants (Paton, 1993). Our observations showed that honeybees (*A. mellifera*) visited a majority of flowers within an inflorescence by moving along the inflorescence and probing one flower after another. The three bumblebee species exploited slightly lower percentage of flowers, while small solitary bees and Diptera usually probed only a few flowers per inflorescence (Fig. 7B). Different pollinators thus have a different potential for geitonogamous pollination, because pollen from the previously visited plant is deposited mostly on stigmas of the first few flowers and receipt of foreign pollen exponentially decreases in each successive flower visit within an inflorescence (Thomson & Plowright, 1980; Gerber, 1985; Morris et al., 1994). High levels of geitonogamous pollination may negatively affect plant fitness (Gerber, 1985; Waser & Price, 1991; de Jong, Waser & Klinkhamer, 1993; Ruane et al., 2013). In our population of *S. verticillata*, we would expect higher level of geitonogamous pollination in inflorescences higher above ground because of shifts in the flower visitor community (see Fig. 7), perhaps also in individual whorls higher within an inflorescence. However, proportional seed set at the scale of entire inflorescences and individual whorls increased with height, as a consequence of higher total visitation rate.

Inflorescences which are more attractive for pollinators may also attract higher numbers of florivores and seed predators (Sletvold & Grindeland, 2008). Seed set is thus driven by a balance between mutualistic and antagonistic interactions with flower visitors (Ehrlén, Käck & Ågren, 2002; Schlinkert et al., 2016). We did not notice any conspicuous evidence of florivory during observations of flower visitors or seed predation when processing seeds of *S. verticillata*. However, in other plant species, florivory and seed predation can have a large negative effect on plant fitness (Ruane, Rotzin & Congleton, 2014). For example, Schlinkert et al. (2016) found that abundance of both pollinators and florivores increased with plant height and mutualistic and antagonistic interactions had contrasting effects on the number of seeds leading to seet set being independent of plant height. The role of inflorescence height may thus be species-specific and context-dependent, as shown also by our observations of inflorescence visitation in short and tall vegetation.

## Conclusion

In conclusion, we experimentally demonstrated that both total flower visitation and the composition of the community of insect visitors changed with the vertical position of inflorescences in three common plant species growing in a dry grassland. Moreover, we found that the dependence of visitation rate on inflorescence height was mediated by the height of the surrounding vegetation. In one species, *S. verticillata*, we also observed increased seed set with inflorescence height, which supports the hypothesis of selection for increased inflorescence height. Overall, we detected pronounced vertical stratification of plant–pollinator interactions at a scale of mere decimetres in a temperate grassland.

## Supplemental Information

10.7717/peerj.4998/supp-1 Supplemental Information 1 Table S1. Complete inflorescence visitation data from the experiment on the role of inflorescence height and the height of the surrounding vegetation in *Centaurea scabiosa* and *Inula salicina* The number of inflorescence visits on individual plants in experimental transects surrounded by short or tall vegetation is shown for each flower visitor species or group.

10.7717/peerj.4998/supp-2 Supplemental Information 2 Table S2. Total visitation of inflorescences of *Salvia verticillata* depending on inflorescence height. Total number of visitors arriving in each inflorescence during one recording period of approximately 30 minutes is provided for three ramets per plant differing in inflorescence height. Total number of flower visits during the recording period is also provided because most visitors probed multiple flowers per inflorescence visit.

10.7717/peerj.4998/supp-3 Supplemental Information 3 Table S3. Records of individual visits of inflorescences of *Salvia verticillata* Identity of a flower visitor and the number of flowers probed is provided for each inflorescence visit on individual plants. Three ramets per plant were observed and can be distinguished by different inflorescence height.

10.7717/peerj.4998/supp-4 Supplemental Information 4 Table S4. Seed set in individual ramets of *Salvia verticillata* in relation to inflorescence height. The number of flowers and the number of developed seeds was counted in individual whorls in inflorescences of each *Salvia verticillata* plant. Three ramets differing in inflorescence height were used in each plant. Inflorescences were composed of the main stem and usually 1-2 side branches; which were counted separately.

## Additional Information and Declarations

### Data Availability

The following information was supplied regarding data availability: The raw data are provided in the Supplemental Tables.
